# Research Progress into the Biological Functions of IFITM3

**DOI:** 10.3390/v16101543

**Published:** 2024-09-29

**Authors:** Qian Xie, Liangliang Wang, Xinzhong Liao, Bi Huang, Chuming Luo, Guancheng Liao, Lifang Yuan, Xuejie Liu, Huanle Luo, Yuelong Shu

**Affiliations:** 1 School of Public Health (Shenzhen), Shenzhen Campus of Sun Yat-Sen University, Shenzhen 518107, China; xieq59@mail.sysu.edu.cn (Q.X.); liaoxzh8@mail2.sysu.edu.cn (X.L.); huangb63@mail2.sysu.edu.cn (B.H.); luochm7@mail.sysu.edu.cn (C.L.); liaogch5@mail2.sysu.edu.cn (G.L.); yuanlf6@mail2.sysu.edu.cn (L.Y.); liuxj89@mail2.sysu.edu.cn (X.L.); 2 Chinese Academy of Medical Sciences & Peking Union Medical College, Beijing 100730, China; wanglliang3@mail2.sysu.edu.cn; 3 Division of HIV/AIDS and Sex-Transmitted Virus Vaccines, Institute for Biological Product Control, National Institutes for Food and Drug Control (NIFDC), WHO Collaborating Center for Standardization and Evaluation of Biologicals NHC Key Laboratory of Research on Quality and Standardization of Biotech Products and NMPA Key Laboratory for Quality Research and Evaluation of Biological Products, Beijing 102629, China; 4 Key Laboratory of Tropical Disease Control (Sun Yat-sen University), Ministry of Education, Guangzhou 510080, China; 5 Key Laboratory of Pathogen Infection Prevention and Control (MOE), State Key Laboratory of Respiratory Health and Multimorbidity, National Institute of Pathogen Biology, Chinese Academy of Medical Sciences & Peking Union Medical College, Beijing 102629, China

**Keywords:** IFITM, IFITM3, structure, function

## Abstract

Interferon-induced transmembrane proteins (IFITMs) are upregulated by interferons. They are not only highly conserved in evolution but also structurally consistent and have almost identical structural domains and functional domains. They are all transmembrane proteins and have multiple heritable variations in genes. The IFITM protein family is closely related to a variety of biological functions, including antiviral immunity, tumor formation, bone metabolism, cell adhesion, differentiation, and intracellular signal transduction. The progress of the research on its structure and related functions, as represented by IFITM3, is reviewed.

## 1. Introduction

The interferon-induced transmembrane protein (IFITM) family consists of small interferon-induced transmembrane proteins with a molecular weight of approximately 17 kDa. Human cells express at least five members of this family: IFITM1, IFITM2, IFITM3, IFITM5, and IFITM10. IFITM5 is exclusively expressed in bone cells, whereas the function of IFITM10 remains unclear. The other three IFITM proteins are broadly expressed across various cell types in the human body and show a strong response to interferon activation. Currently, the primary focus is on IFITM’s broad antiviral functions across the IFITM family, where IFITM3 exhibits the strongest antiviral activity, whereas IFITM5 and IFITM10 lack such activity [1,2]. IFITM3 has demonstrated efficacy against a broad spectrum of almost all enveloped viruses, including dengue virus, influenza A Virus (IAV), H1N1, Zika virus, coronaviruses, hepatitis C virus, West Nile virus, vesicular stomatitis virus (VSV), and human immunodeficiency virus (HIV), HCoV-229E, and MERS-CoV, as well as SARS-CoV-2 [3,4,5,6,7,8,9,10]. In addition to its antiviral function, extensive research on IFITM3 has focused on its role in immune regulation, tumor development, and progression, and its effects on the nervous system [11,12,13]. Numerous studies have identified associations between several single nucleotide polymorphisms (SNPs) located within the coding region of the IFITM3 gene and a range of diseases [13,14,15,16,17]. Specifically, the *IFITM3* SNP rs12252 is of great interest. When the T allele is replaced by the C allele, this variation is predicted to alter the splice acceptor site. This leads to the deletion of 21 amino acids from the N-terminus of IFITM3 (NΔ21) [16,18]. The deletion of these 21 amino acids compromises the capacity of IFITM3 to effectively block virus entry into host cells [19]. Previous studies have found that this SNP is associated with the severity of influenza infection [20]. Moreover, the latest research on the analysis of a cohort of COVID-19 patients suggests that individuals carrying the rs12252 C allele in the *IFITM3* gene may be more susceptible to SARS-CoV-2 infection [21,22].

## 2. Discovery of the *IFITM* Genes

The *IFITM* gene was first identified in adult neuroblastoma cells following treatment with interferons [23]. Its promoter contains one or more interferon-stimulated response elements (ISREs), thereby enabling induction by type I, type II, or type III interferons [24]. Current research on IFITMs has predominantly focused on humans and mice. Humans possess five *IFITM* genes, all located on chromosome 11, while mice have seven IFITM genes. Humans and mice share five IFITM members, exhibiting homology ranging from 77.46 to 87.54%.

IFITM family proteins play a defense role against viral infection by inhibiting viral membrane fusion with host cells. Alber et al. [25] first described the function of IFITM1 as an inhibitor of the vesicular stomatitis virus (VSV) infection. Brass et al. [26] identified the antiviral activity of the IFITM1, IFITM2, and IFITM3 proteins against IAV, dengue virus, and flaviviruses (e.g., West Nile Virus) using an siRNA interference screening. Subsequent studies have suggested that IFITM proteins exhibit broad-spectrum antiviral effects against various enveloped and non-enveloped viruses, including influenza viruses, dengue viruses, Ebola viruses, hepatitis B viruses, coronaviruses, adenoviruses, cytomegalic viruses, arboviruses, murine leukemia viruses, and alphaviruses (Table 1). Due to the comprehensive research on IFITM3, we will focus on summarizing its research progress.

## 3. Molecular Evolution of IFITM3 Protein

**Figure 1:**
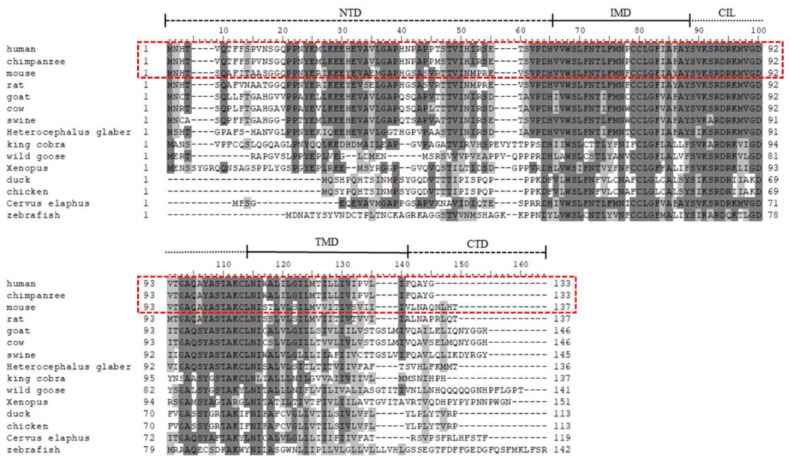
Alignment of the amino acid sequences of the IFITM3 protein with the red dashed lines for humans, chimpanzees, and mice. (IFITM3 are derived from avian, non-avian reptiles and amphibians). The shade of color represents the level of sequence consistency, with dark gray indicating complete sequence consistency.

Homologous *IFITM* family genes have been identified across a multitude of species, including mammals, marsupials, birds, fish, and reptiles, suggesting a significant conserved role for IFITM proteins. Regions are displaying a high degree of gene sequence homology among human IFITM superfamily genes, with the coding regions displaying up to 88% similarity [44]. Numerous studies have dissected the evolution and function of IFITM proteins across various species, revealing sites under positive selection. Scheben et al. found that three codon sites in the IFITM intramembrane domains (IMDs) and transmembrane domain (TMD) show evidence of positive selection [45,46]. Smith et al. found that lFlTM3 is expected to have fewer revealed sites under positive selection in chickens and ducks. They used CODEML to predict two additional sites under persistent positive selection in the N-terminal region of IFITM3 [47]. In comparison to the analysis by Smith et al., the analysis by Bassano et al. identified far fewer positively selected sites, detecting only two such sites in chicken IFITM3 [48]. *IFITM3* shows recurrent gene duplication and divergence during primate evolution [49]. Human and mice IFITM genes are evolutionarily related but do not exhibit one-to-one orthologs. All the IFITM3 genes are derived from avian and non-avian reptiles and amphibians that had almost the same NTD, IMD, conserved intracellular loop (CIL), C-terminal domain (CTD), and TMD (Figure 1).

## 4. Structure of IFITM3 Protein

**Figure 2:**
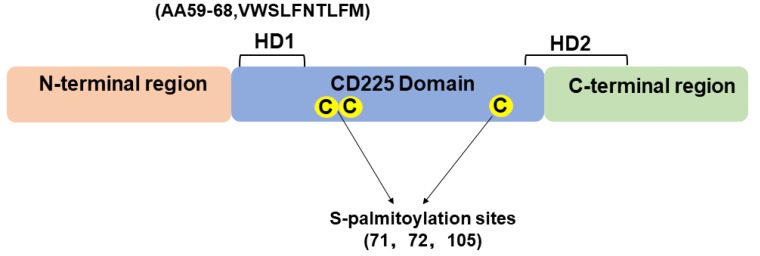
Molecular domains of the IFITM family proteins.

The IFITM family proteins share a conserved CD225 structural domain with highly variable regions at both termini. The CD225 domain contains an intact transmembrane region with two S-palmitoylation sites and a partial transmembrane region located at the C-terminus (Figure 2) [44,50]. IFITM proteins exhibit a common topological architecture, including an N-terminus and a C-terminus, two transmembrane domains, and a short conserved cytoplasmic domain. Notably, the N-terminal tail extends longer than the C-terminal tail [51,52]. The two transmembrane regions are a conserved and hydrophobic domain (HD), referred to as the amphipathic helix. Chesarino et al., using a bioinformatic approach, predicted IFITM3 secondary structures and identified a highly conserved, short amphipathic helix within a hydrophobic region of IFITM3, and they showed that this helix and its amphipathicity are required for the IFITM3-dependent inhibition of influenza virus, Zika virus, vesicular stomatitis virus, Ebola virus, and human immunodeficiency virus infections [53]. Both amphipathic helices of IFITM3 are S-palmitoylated, with three transmembrane domain-proximal cysteine residues at specific positions serving as potential sites of S-palmitoylation [54]. The S-palmitoylation site is a crucial post-translational modification (PTM) for the stabilization of IFITM proteins and their association with antiviral activity [54,55]. As a member of the IFITM family, IFITM3 possesses a similar structural conformation, with a molecular weight of 15 kDa. It is heavily regulated by post-translational modifications. S-palmitoylation is a primary PTM that contributes to the stabilization of IFITM proteins. Moreover, previous studies have reported three additional PTMs that negatively modulate the antiviral activity of IFITM3: ubiquitination at one or more of four lysine residues [56], methylation on K88 [57], and phosphorylation on Y20 [55,58]. IFITM3 also forms homo- and hetero-oligomers [59].

**Figure 3:**
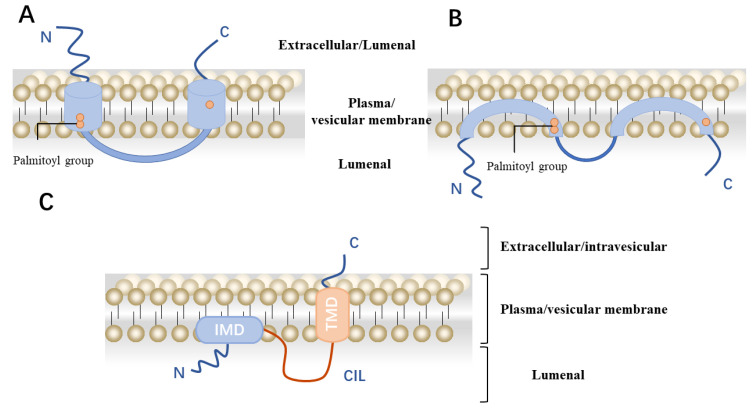
Schematic representation of the possible topology of the IFITM family. (**A**) Schematic diagram of the IFITM family protein type III transmembrane protein topology. (**B**) Schematic representation of the intramembrane topology of IFITM family molecules. (**C**) Schematic representation of IFITM3 protein type II transmembrane. IMD, intramembrane domain. TMD, transmembrane domain.

The structure of IFITM remains unclear, but there are three different hypotheses about the structure of IFITM currently. The first hypothesis was deduced through analysis of the protein sequence, which are type III transmembrane proteins with two transmembrane regions. The N-terminal and C-terminal ends are located in the extracellular space or the lumen of the endoplasmic reticulum (ER), while the protein ring structure is situated intracellularly (Figure 3A) [51]. The second hypothesis arises from initial research findings indicating that antibodies binding to Leu-13 at the N-terminus of IFITM1 can induce lymphocyte aggregation, suggesting an extracellular localization for this region. Furthermore, flow experiments have corroborated these findings by identifying the N-terminal epitope of IFITM at the cell surface. However, subsequent studies have added complexity to this understanding. Intracellular ubiquitinase has been found to modify the N-terminal ubiquitination site Lys-24 of IFITM3, suggesting an intracellular location for the N-terminus of IFITM3, with the NTD module lacking N-linked glycosylation [49,60]. Moreover, phosphorylation of Tyr-20 at the N-terminus of IFITM3 is crucial for its endocytosis into endosomes or lysosomes, with Fyn identified as the corresponding kinase, highlighting the N-terminus’s interaction with cytoplasmic enzymes [60]. Additionally, in murine IFITM1, a cysteine in the second transmembrane region near the C-terminus is palmitoylated, suggesting a lumenal conformation for the C-terminus of IFITM1. Incorporating these findings, a model proposing an endosomal topology for the IFITM molecule is presented in Figure 3B [61]. 

Furthermore, studies concentrating on the topological structure of mouse IFITM3 have revealed intriguing insights. It has been observed that the N-terminus of IFITM3 can be detected with antibodies; however, this conformation appears to be contingent upon the cell type. Specifically, only a small proportion of cell membrane proteins exhibit this phenotype. Conversely, the C-terminus of IFITM3 constitutes a significant portion of the extracellular membrane in this conformation. Further investigation has pinpointed the ER retention signal of IFITM3 to the C-terminal end. The presence of a KDEL sequence at this terminus enables IFITM3 to be retained within the ER, with the orienting sequence positioned in the lumen of the ER [51]. Evidence supporting the degradation of the C-terminal sequence within lysosomes substantiates the hypothesis that the C-terminal tail structure is localized to the lysosomal lumen. Furthermore, when IFITM3 is expressed in isolation, TM2 fulfills the role of a signaling anchor sequence for IFITM3. Collectively, this experimental evidence suggests the existence of a third structural conformation for IFITM proteins. Consequently, it is proposed that IFITM3 adopts a type II transmembrane protein configuration [62]. In this model, TM1 is categorized as the intramembrane transmembrane sequence, while TM2 constitutes the complete transmembrane sequence (Figure 3C) [63].

## 5. Biological Functions of IFITM3

### 5.1. Antiviral Effects of IFITM3

IFITM3 exhibits a broad-spectrum inhibitory activity against a variety of viral infections. Brass et al. [26] demonstrated that inhibiting IFITM3 expression using small interfering RNA or shRNA resulted in increased susceptibility to IAV infection. Consistently, Everitt et al. [18] and Bailey et al. [64] found that *Ifitm*3^−/−^ mice infected with the IAV showed higher morbidity and mortality compared to wild-type (WT) mice. Furthermore, Everitt et al. discovered that the *Ifitm*3^−/−^ mice infected with the IAV developed more severe parenchymal lung damage and viral pneumonia [65]. The exogenous expression of IFITM1, IFITM2, or IFITM3 inhibited the replication of various viruses, including influenza A virus (IAV), West Nile virus, dengue virus, yellow fever virus, SARS-CoV-2, and others [26,66,67]. 

**Figure 4:**
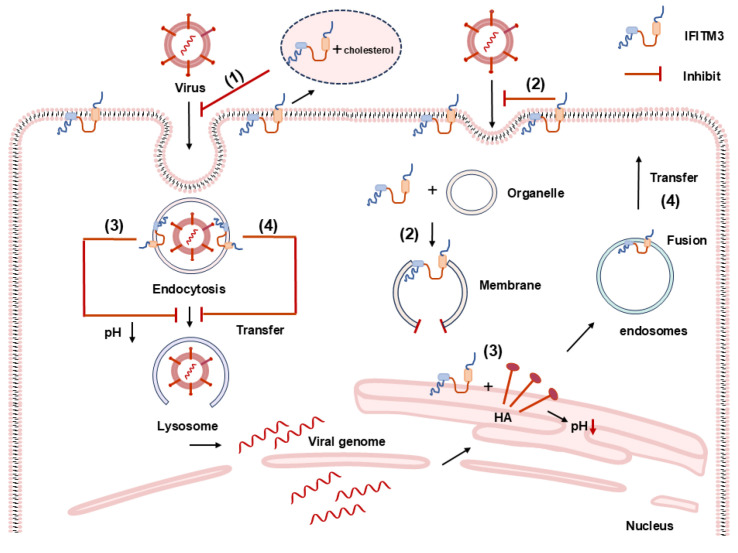
Possible antiviral mechanisms of IFITM3. (1) The amphipathic helical peptide (AH peptide) of IFITM3 may interact directly with cholesterol analogs to inhibit the formation of membrane fusion, thereby preventing viral entry. (2) IFITM3 may inhibit the fusion of virus and host cell membranes both by decreasing cell membrane fluidity and by stabilizing the cytoplasmic layer of the endosomal membrane to restrict viral entry from the intracellular compartment. (3) IFITM3 may interact with influenza virus haemagglutinin (HA) to reduce the optimal pH for membrane fusion, which in turn affects virus replication. (4) IFITM3 located in the lysosomal membrane may inhibit viral entry by disrupting transport processes in endosomes.

IFITM3 utilizes at least four distinct mechanisms to inhibit virus replication. Firstly, it disrupts lipid homeostasis within cells by modifying the properties of the intraluminal vesicles and endosomal membrane [68]. Lipid membranes, forming the bilayer of the cell membrane, serve as barriers that tightly regulate the entry and exit of numerous viruses. Cholesterol, essential for the integrity of lipid raft membranes, endosomal compartments, and other organelles, plays a pivotal role in this process [68]. IFITM3 has been found to antagonize the function of VAPA-OSBP, thereby interfering with intracellular cholesterol homeostasis. This interference leads to an increase in endosomal cholesterol levels, consequently inhibiting vesicle fusion and virus entry [68]. Furthermore, Rahman et al. [53,69,70] found that IFITM3 inhibits the entry of IAV by acting through the amphipathic helix (AH) in its IMD. The AH peptide of IFITM3 directly engages with the cholesterol analog NBD-cholesterol, facilitating the inhibition of membrane fusion pore formation (Figure 4(1)). This, in turn, disrupts the entry process of the influenza virus. 

The second antiviral mechanism involves hindering the fusion process between viral and host cell membranes (Figure 4(2)). This inhibition may occur through various means, such as reducing membrane fluidity and altering spontaneous curvature [71,72]. The experiment of adding oleic acid (OA) has provided evidence that the presence of IFITM may block virus–membrane hemifusion by making the spontaneous curvature of the outer leaflet of the plasma membrane more positive [72]. The experiment using a hydrophobic fluorescent probe has shown that the expression of IFITM increases the lipid packing order of the cell membrane, which reduces the membrane fluidity and thereby inhibits the fusion between the virus and the host cell membrane [72]. Research conducted by Desai et al. [70,71,73] yielded contrasting results. They observed that an excess of cholesterol in late endosomes of IFITM3-expressing cells inhibited IAV entry, and IFITM3 prevented influenza virus entry into the host cell by blocking the forming of the fusion pore. This suggests that IFITM3 may stabilize the cytoplasmic leaflets of endosomal membranes, either directly or indirectly, by modulating the physical properties of the cell membrane (Figure 4(2)). These findings suggest that IFITM may restrict viral entry from a subset of intracellular compartments [71]. 

The third antiviral mechanism pertains to the modulation of pH within the vesicular environment, consequently retarding the acidification rate of endosomes (Figure 4(3)). Enveloped viruses, such as IAV and HIV, often necessitate passage through a sequence of transport vesicles, including early and late endosomes, to facilitate entry into host cells. Studies have shown that IFITM3 can significantly impede the fusion of viral envelopes with the cellular or endosomal membranes, thereby sequestering viral particles within the endocytic pathway. This entrapment culminates in the convergence with lysosomes, where the particles are degraded by a suite of enzymatic processes and subsequently presented to the cell surface via the major histocompatibility complex class I (MHC-I) pathway. Furthermore, non-enveloped viruses, like Reoviruses, utilize the endosomal pathway for cellular entry, a process that IFITM3 has been shown to inhibit. Anafu et al. demonstrated through comparative analyses that cells overexpressing IFITM3 harbored substantially reduced viral loads compared to control cells, suggesting that elevated IFITM3 expression can efficaciously preclude the entry of reoviruses into host cells [74]. Moreover, research has established that the release of the reovirus nucleocapsid is contingent upon the activity of cellular cathepsins, which are acid-dependent proteases that exert their function upon a sufficient decrease in pH. IFITM3 has been demonstrated to modulate the transmembrane ion exchange between the endosome and the cytoplasm, consequently retarding the pH alteration rate. This modulation subsequently inhibits the cathepsin-mediated degradation of the Reovirus capsid protein, leading to the entrapment of the Reovirus genome within the endosome and preventing its release. Consequently, this mechanism impedes the progression of viral infection.

The fourth antiviral mechanism involves influencing the intracellular transport of endosomal vesicles (Figure 4(4)). Studies have demonstrated that IFITM3 can localize to the membranes of nuclear endosomes and lysosomal compartments, where it co-localizes with proteins such as Rab7, CD63, and lysosome-associated membrane protein 1 (Lamp1). These compartments are crucial sites where endocytosed vesicles fuse with viral particles within the host cell and facilitate endosome-to-lysosome transport. The possible antiviral mechanism is that it may prevent virus entry by altering rates of virus–endosome fusion [72] and/or accelerating the trafficking of endosomal cargo to lysosomes for destruction [49,75]. The distinct sub-localization of IFITM1, 2, and 3 proteins within the cell may contribute to their varied activities in inhibiting the entry of different viruses into their specific fusion sites, particularly through cell fusion driven by syncytia [60,76]. The latest research has found that the CD225 region of IFITM3 contains a SNARE-like motif, which can block homotypic late endosome fusion, diverting the entering virus to the lysosome and accelerating the degradation of viral particles [77].

In addition to the four widely recognized antiviral mechanisms mentioned above, other studies provide new insights. *IFITMs* are interferon-stimulated genes (ISG), which can indirectly inhibit viral replication or infection by regulating the expression of Rab5 and Caveolin-1 in endosomal compartments. In addition, IFITM proteins enhance their antiviral effects indirectly through the activation of the IFN-β signaling pathway triggered by MDA5, and the N-terminal domain of IFITM2 plays an important role in the antiviral activity and activation of IFN-β [78]. This reveals a feedback regulatory pathway between IFITM proteins and IFN-β [78]. This feedback regulatory pathway may play a role in a variety of infections and immune-related diseases [79].

### 5.2. Immunomodulatory Effects of IFITMs

Researchers have investigated the role of IFITMs in adaptive immunity. The expression levels of IFITM1 and IFITM3 are upregulated in a variety of mammalian immune cells upon activation, including macrophages, dendritic cells, T cells, and B cells. IFITM family proteins exert an influence on the morphology and function of cell membranes, thereby affecting cellular susceptibility to viruses and modulating immune responses [80,81]. For instance, IFITM3 enhances cell-mediated immune responses by augmenting antigen presentation in dendritic cells. Furthermore, IFITM3 modulates cellular responses to interleukin (IL)-6 and IL-10, thereby influencing the profile of cellular immune responses [11,82], and it promotes MyD88-dependent, TLR-mediated IL-6 production following exposure to cytomegalovirus (CMV) [83,84]. IFITM3 also restricts IL-6 production by targeting Nogo-B in response to influenza and SARS-CoV-2 [84]. Additionally, IFITM is implicated in the B-cell co-receptor CD19/CD21/CD81 complex, which facilitates antigen-specific B-cell activation while reducing cell-surface L-selectin expression [17,85,86]. The interaction of mouse IFITM3 with tetraspanin CD9 and CD81 proteins was described [85]. Depletion of IFITM3 in T cells led to reduced surface CD3 levels and inhibition of TCR signaling [17]. Thus, the direct interaction between CD81 and IFITM may extend its function from antiviral activity to immunomodulatory activity. Furthermore, kinases such as BCR-ABL3 and LYN can phosphorylate IFITM [17], promoting endosome localization and changing to the plasma membrane, where it is involved in BCR signaling and associated malignant transformation [17,87]. IFITM also regulates cytokine production. IFITM3 suppressed the cytokine storm associated with respiratory virus infection. In IFITM3-deficient mice, increased inflammatory and apoptotic responses, along with pathologically activated NK cells in the lungs and spleens, have been observed [65]. A similar effect has been noted in patients infected with H7N9 carrying the IFITM3 rs12252-C/C genotype, an SNP affecting antiviral function. In such cases, patients exhibited higher levels of plasma cytokines, especially IL-6, IL-8, and MIP-1β, which are associated with poor clinical outcomes [88].

**Figure 5:**
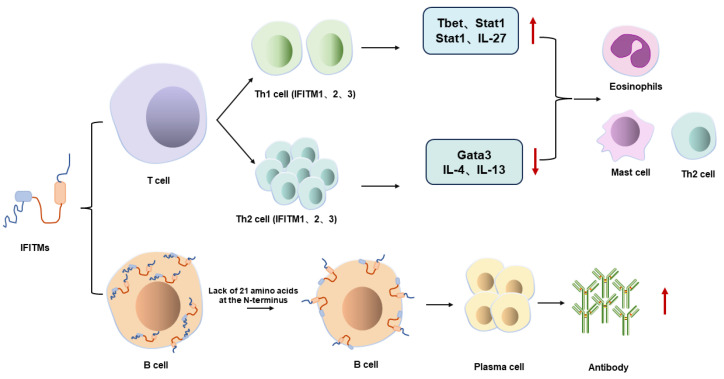
Possible mechanisms of IFITMs immunomodulatory effects. The expression of IFITMs was upregulated in a variety of immune cells upon activation. Th1 cells enhanced the immune function of eosinophils, macrophages, and Th2 cells by upregulating Tbet, stat1 IL-27, etc. Th2 cells facilitated this process by downregulating Gata3, IL-4, and IL-13. In addition, IFITM3 with a 21-amino-acid deletion at the N-terminus on the surface of B cells promotes antibody production by plasma cells to enhance humoral immunity. “↑”, increase; “↓”, decrease.

IFITM3 is expressed in lymphocytes of both murine and human origin, and numerous studies have demonstrated its association with T cell receptor (TCR) signaling complexes [89,90,91]. Furthermore, IFITM3 is likely to play a role in T cell differentiation, as evidenced by gene and protein expression studies indicating its significant impact on T cell function [11,80,92,93]. The expression of IFITM3 is regulated by the TCR signaling pathway, with its expression rapidly downregulated in naïve CD4^+^ T cells within 24 h following anti-CD3/CD28 activation under helper T cell type 0 (Th0), Th1, and Th2 culture conditions [11]. In contrast, Western blotting (WB) analysis revealed that IFITM3 protein expression on naive CD8^+^ and CD4^+^ T cells was upregulated by day 3 post T cell activation via anti-CD3/CD28 ligation, and this upregulation occurred independently of interferon signaling [81] (Figure 5). The differences in IFITM3 protein expression patterns may arise from variations in the intensity of activation signals or differences in the rates of flip-flopping and ubiquitination of the IFITM3 protein during TCR activation [94,95,96]. Consequently, while IFITM3 gene expression initially declined, IFITM3 protein levels subsequently increased. Furthermore, IFITM3 is regulated by Hedgehog (Hh)-mediated transcription in mouse CD4^+^ T cells [92].

In addition, IFITM3 is intimately associated with immune-related diseases, including allergic reactions and inflammation-related diseases [11]. In individuals with atopic dermatitis, IFITM3 expression was found to be upregulated in lesional skin cells compared to non-lesional skin cells from the same individuals; however, the mechanism underlying this upregulation requires further investigation [97]. Similarly, increased IFITM3 expression has been observed in the inflamed mucosa of patients with ulcerative colitis and Crohn’s disease [98,99]. Genetic polymorphisms in IFITM3 have been associated with susceptibility to ulcerative colitis [100,101]. Furthermore, the absence of IFITM3 has been correlated with the exacerbation of chemically induced colitis, increased infiltration of macrophages and effector T cells into the colon’s lamina propria, and a shift in the differentiation of CD4^+^ T cell towards the Th17 subtype [102]. Additionally, IFITM3 plays a role in regulating cytokine signal transduction pathways. For instance, it is implicated in the IFN receptor signaling pathway, which is dependent on clathrin-mediated endocytosis for internalization [103]. Therefore, the presence of IFITM3 on late endosomal membranes may modulate the IFN signaling pathway. These findings highlight the multifaceted role of IFITM3 in immune regulation and its implications in immune-related diseases. IFITM3 also plays a role in regulating the humoral immune response. An epidemiological study revealed that compared to rs12252-T/T carriers, individuals with the rs12252-C/C genotype of IFITM3 exhibited lower levels of hemagglutination inhibition (HI) antibody responses against H1N1, H3N2, and B viruses following trivalent inactivated influenza vaccine (TIV) immunization. This suggests an association between IFITM3 rs12252 and immune response [104]. Additionally, Lei et al. [105] showed that deletion of the IFITM3 gene led to reduced levels of HI, microneutralization (MN), and IgG antibodies against H1N1, H3N2, and B/Victoria viruses in mice after TIV immunization, with a delayed peak of antibody response. This effect may be attributed to the disruption of the balance between Blimp1 and BCL6, resulting in abnormalities in the transcriptional network regulating germinal center B cell plasmablast differentiation. Furthermore, Xie et al. [76] observed that after booster immunization with quadrivalent inactivated influenza vaccine (QIV), mice carrying the IFITM3 rs12252-C/C genotype with an N-terminal truncation of 21 amino acids (NΔ21) exhibited higher levels of HI, MN, and IgG antibodies against influenza viruses compared to WT mice. This enhanced humoral immune response may be mediated by the NΔ21 protein, which potentially migrates to prevent the degradation of CD81, thereby facilitating antibody production (Figure 5). These findings highlight the role of IFITM3 in modulating the humoral immune response to viral infections and vaccinations.

### 5.3. The Role of IFITM3 in Tumorigenesis

IFITM3 is frequently overexpressed in various tumor tissues, exhibiting the highest expression levels among the IFITM family in both normal and tumor tissues [11,17,106]. While the precise mechanisms and effects of IFITM3 in tumor immunity are not yet fully understood, its high expression in tumor cells suggests tumorigenic properties [107]. It remains unclear whether IFITM3 overexpression occurs solely in transformed cancer cells, matrix cells, or both, and the underlying mechanism remains elusive. IFITM3 is known to regulate tumor occurrence and development by modulating cancer cell proliferation, cell cycle progression, and apoptosis. Research indicates that IFITM1 functions as a negative regulator of cell proliferation, inducing cell cycle arrest via a p53-dependent mechanism [91,108,109]. However, this cell cycle arrest mechanism appears to be dysfunctional in IFITM3, as evidenced by its overexpression in oral squamous cell carcinoma (OSCC) cells, where it potentially modulates the CCND1-CDK4/6-pRB axis to facilitate OSCC cell proliferation [110]. Additionally, IFITM3 regulates cell migration, invasion, and metastasis by activating signaling pathways such as the PI3K/Akt/mTOR pathway, which plays a pivotal role in epithelial–mesenchymal transition (EMT) [111]. Furthermore, IFITM1, IFITM2, and IFITM3 exert an impact on the p38/MAPK signaling pathway, resulting in the upregulation of extracellular matrix metalloproteinases, MMP2, and MMP9, which are essential for cell migration by remodeling the extracellular matrix [112,113,114,115,116]. IFITM3′s effect on MAPK pathway activation is also associated with TGFβ/Smad signaling transduction. Through direct interaction with Smad4, IFITM3 acts as a regulatory molecule of the TGFβ/Smad/MAPK signaling pathway, promoting EMT, cell proliferation, migration, and bone metastasis in prostate cancer [117]. In the same way, TGFβ has been shown to stimulate IFITM3 expression [118,119]. Moreover, IFITM proteins have been implicated in angiogenesis, a process integral to tumor development. The upregulation of IFITM proteins in endothelial progenitor cells influences the vascular lumen, with endothelial cells deficient in IFITMs exhibiting an inability to form lumens properly to form lumens normally [120]. IFITM proteins are also implicated in tumor progression and have been identified as molecule targets that can influence the efficacy of anti-cancer therapies, including radiotherapy, chemotherapy, and endocrine therapy [121,122,123,124]. In breast cancer, the expression of IFITM3 is positively correlated with the development of resistance aromatase inhibitors, which is associated with decreased activities of STAT1 and STAT2, leading to reduced p21 expression through a mechanism that is independent of p53 [125,126]. Additionally, IFITM proteins may serve as both prognostic and detection markers for a range of solid tumor types and hematologic malignancies. The ability of IFITM3 to confer spheroid-forming upon various cancers suggests its potential role in the maintenance of cancer stem cells [110,115,127]. Thus, IFITMs play diverse and critical roles in tumorigenesis and tumor progression, making them potential targets for cancer therapy and prognostic markers for cancer detection and treatment evaluation.

### 5.4. The Other Biological Functions of IFITM3

IFITM3 has emerged as a key player in the regulation of neurodegenerative diseases. Studies have demonstrated its involvement in various aspects of neurodevelopment and neuropathological damage. For instance, neonatal treatment of mice with poly I: C, a toll-like receptor 3 inducer of the innate immune response, significantly increased IFITM3 levels in hippocampal astrocytes. This led to long-term brain dysfunction, including cognitive and mood deficits and deficient glutamate release in the hippocampus during adulthood. Notably, neonatal poly I: C-induced neuronal damage was not observed in *ifitm*3^−/−^ mice, indicating a crucial role for IFITM3 in mediating the neurodevelopmental effects of innate immune system activation [128]. Furthermore, IFITM3 has been implicated in the development of Alzheimer’s disease (AD), potentially through its regulation of γ-secretase activity and modulation of amyloid-beta expression in cells [12]. Molecular epidemiological studies have also associated IFITM3 with central nervous system (CNS) pathologies, including schizophrenia [127,128].

## 6. Future Directions in IFITM Research

**Figure 6:**
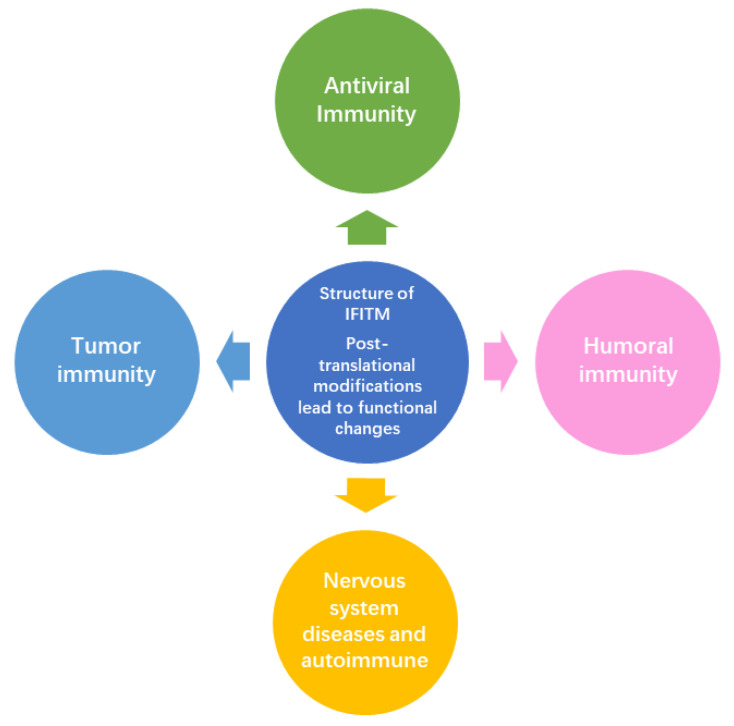
Prospects for future research directions in IFITM.

In summary, IFITM family proteins, as crucial interferon-stimulated immune molecules, play important roles in various biological processes, including cellular immunity, tumor growth, metastasis, and neurodegeneration (Figure 6). Although their structures and mechanisms of action are not fully understood, a growing body of research has elucidated their antiviral and immunomodulatory mechanisms. Despite their evolutionary conservation, minor sequence and structural variations, together with genetic polymorphisms, can significantly influence their function. Moreover, post-translational modifications, oligomerization, and interactions with other proteins add complexity to their study. Future research will focus not only on their antiviral function but also on their roles in autoimmune diseases, anti-tumor immunity, and other immune-related functions. There will be a particular emphasis on understanding their spatiotemporal and cell type-specific expression patterns and elucidating their structures to uncover their full spectrum of functions.

## Figures and Tables

**Table 1:** Antiviral profile of IFITM3.

Inhibited	Resistant [2,27]
orthomyxoviruses (such as IAV [6]), paramyxoviruses (parainfluenza virus [28], metapneumovirus [29], and respiratory syncytial virus [30,31,32]), rhabdoviruses (vesicular stomatitis virus (VSV), flaviviruses (WNV [6], DENV [6], hepatitis C virus (HCV) [33], Zika virus (ZIKV) [34] and yellow fever virus [35]), filoviruses (Ebola virus (EBOV) [3,9] and Marburg virus [3]), poxviruses (vaccinia virus and cowpox virus (CPXV) [36], bunyaviruses (Rift Valley fever virus and La Crosse virus) [37], alphaviruses (chikungunya virus [38], Sindbis virus [39], Semliki Forest virus [39]), lentiviruses (human and simian immunodeficiency viruses) [5,40,41], and coronaviruses (human coronavirus 229E (hCoV-229E) [42], severe acute respiratory syndrome coronavirus (SARS-CoV) [3], Middle East respiratory syndrome coronavirus (MERS-CoV) [4] and SARS-CoV-2 [43])	amphotropic murine leukemia virus, Sendai virus, papillomavirus, cytomegalovirus, adenovirus, and the arenaviruses Lassa virus (LASV), Machupo virus, and lymphocyticchoriomeningitis virus
